# Vitamin D levels are low in adult patients with sickle cell disease in Jamaica and West Africa

**DOI:** 10.1186/2052-1839-14-12

**Published:** 2014-08-16

**Authors:** Bamidele O Tayo, Titilola S Akingbola, Babatunde L Salako, Colin A McKenzie, Marvin Reid, Jennifer Layden, Ifeyinwa Osunkwo, Jacob Plange-Rhule, Amy Luke, Ramon Durazo-Arvizu, Richard S Cooper

**Affiliations:** Department of Public Health Sciences, Loyola University Chicago Stritch School of Medicine, 2160 S. First Ave, Maywood, IL 60153 USA; Department of Hematology, College of Medicine, University of Ibadan, Ibadan, Nigeria; Department of Medicine, College of Medicine, University of Ibadan, Ibadan, Nigeria; Sickle Cell Unit, Tropical Medicine Research Institute, The University of the West Indies, Kingston, Jamaica; Department of Medicine, Section of Infectious Diseases, Loyola University Chicago Stritch School of Medicine, Maywood, IL USA; Aflac Center for Cancer and Blood Disorders Service Comprehensive Sickle Cell Program of Children’s Healthcare of Atlanta, Atlanta, GA USA; Kwame Nkrumah University of Science and Technology, Kumasi, Ghana

**Keywords:** Sickle cell disease, Sickle cell anemia, Vitamin D, 25-hydroxyvitamin D, Adult patients, Tropical Africa, Jamaica, West Africa

## Abstract

**Background:**

Patients with sickle cell disease in the USA have been noted to have lower levels of vitamin D – measured as 25-hydroxyvitamin D (25(OH)D) – compared to controls. Average serum 25(OH)D levels are also substantially lower in African Americans than whites, while population distributions of 25(OH)D among Jamaicans of African descent and West Africans are the same as among USA whites. The purpose of this study was to examine whether adult patients with sickle cell disease living in tropical regions had reduced 25(OH)D relative to the general population.

**Methods:**

We analyzed serum 25(OH)D in stored samples collected from studies in Jamaica and West Africa of adult patients with sickle cell disease and adult population controls.

**Results:**

In samples of 20 Jamaicans and 50 West Africans with sickle cell disease mean values of 25(OH)D were 37% and 39% lower than controls, respectively. Metabolic abnormalities in the absorption and conversion pathways are possible causes for the consistent relative deficiency of 25(OH)D in sickle cell disease.

**Conclusions:**

Low 25(OH)D levels in tropical Africa where the burden of sickle cell disease is highest, deserve further investigation, and a randomized trial is warranted to address efficacy of supplementation.

## Background

Vitamin D receptors are expressed ubiquitously in human tissues suggesting that this hormone has numerous regulatory functions. Other than its role in bone-mineral metabolism, clear evidence that variation in vitamin D measured as 25-hydroxyvitamin D (25(OH)D) influences health outcomes has been difficult to obtain [1]. Several reports from North America have documented lower 25(OH)D levels in patients with sickle cell disease (SCD) compared to age-matched controls [2–6]. A correlation between low 25(OH)D and severity of the course of SCD has also been observed and a recent case report described dramatic improvement in pain symptoms after vitamin D supplementation in a SCD patient with severe osteoporosis and profound vitamin D deficiency [2]. A subsequent pilot randomized trial that enrolled 46 patients with SCD demonstrated a significant reduction in days with pain with improved physical-activity-related quality of life after supplementation with high dose vitamin D [7]. The authors of these reports suggest that low 25(OH)D may contribute specifically to the almost universal problem of chronic daily pain observed in SCD, perhaps by impairing bone health [7].

Serum 25(OH)D levels are strongly influenced by the amount of ultraviolet radiation that passes through the outer layer of the skin and reaches vascular tissue. In turn, the latitude of a person’s residence and the degree of skin pigmentation determine the amount of radiation (i.e., ultraviolet B [UVB] to which these tissues are exposed. Dietary sources of vitamin D generally make only a small contribution to body stores. 25(OH)D levels are further influenced by genetic variation in receptors and other components of this biological system [8–11]. It is generally believed that strong evolutionary selection – working through the metabolic effects of vitamin D - accounts for the clinal variation in skin color seen in human populations in relation to latitude [12–14]. Other known factors that influence 25(OH)D include age, adiposity and some chronic medical conditions (e.g., chronic kidney disease, use of steroids, etc.).

The underlying basis for the relative deficiency of 25(OH)D among individuals of African descent is unknown, but could reflect absorption or endogenous metabolic abnormalities. To our knowledge, no reports have yet examined 25(OH)D levels in SCD patients of African heritage living near the equator. In this report we confirm lower levels among patients with SCD in tropical environments.

## Methods

### Study subjects

West African SCD patients were recruited from the clinics at the University College Hospital in Ibadan, Nigeria [15]. A random sample of patients were approached and asked to participate without regard to clinical status. Only patients in steady state were recruited and included in the present study. Because clinical exam of SCD patients at the College Hospital included laboratory analyses, some data on markers of renal or liver function such as creatinine, urea, aspartate transaminase, and alanine transaminase generated at the University College Hospital Laboratory as part of routine clinical workup were available on the SCD patients. Population controls were drawn from a population-based cohort study of energy expenditure from Ghana [16]. Nigeria and Ghana are in the same geographical region in West Africa (Ghana's latitude and longitude is 8° N and 2° W, and Nigeria's latitude and longitude is 10° N and 8° E). There were no reported current uses of vitamin D supplements among the SCD patients and the population controls at the time of subject recruitment. Recruitment of SCD patients and controls took place over multiple years and across both dry and wet seasons.

For the Jamaican SCD patients, specimens were chosen from stored plasma from patients being followed at the Sickle Cell Clinic at the University of the West Indies, Mona, Kingston, Jamaica. Apart from being at steady state, no criteria were applied to sample selection. Population controls were drawn from a second population-based cohort study of energy expenditure from Jamaica; this study included similar numbers of men and women [16]. Both the SCD cohort and the population-based cohort studies were done across seasons and spanned multiple years. Because samples for the Jamaican SCD patients had been drawn and stored for intervals up to 10 years, we therefore performed assessment of reliability of stored samples by analyzing duplicate samples from different follow-up years to assess possible effect of storage on serum 25(OH)D. The correlation between duplicate assays was 0.8 and the absolute mean difference 9.0 nmol/l; this is in agreement with reports that stability of serum 25(OH)D is unaffected by multiple freeze-thaw cycles or long-term storage [17, 18].

### Anthropometric measurements

Anthropometric measurements in all study samples were performed and recorded by trained research staff. Information on age, height and body weight on all study subjects in the four samples were obtained by use of standardized physical exams. Height was measured using a stadiometer consisting of a steel tape attached to a straight wall and a wooden headboard. The headboard was positioned with the subject shoeless, feet and back against the wall, and head held in the Frankfort horizontal plane and measurement taken to the nearest 0.1 cm. Body weight was measured to the nearest 0.2 kg on calibrated electronic scales. Body mass index (BMI) was calculated as weight in kilograms divided by the square of height in meters.

### Laboratory assays

All measurements were made at outpatient clinics located in the communities. Standardized protocols were used for blood sampling. Specimens were immediately processed to separate plasma and then frozen. Frozen plasma samples from both West African and Jamaican study sites were shipped to and stored in Chicago from where they were sent to the Department of Laboratory Medicine at the University of Washington for vitamin D analysis. The liquid chromatography-tandem mass spectrometric assay of vitamin D metabolites was performed as described previously [19, 20]. The calibration of the assay was verified using the National Institute of Standards and Technology standard reference material (SRM) 972. Interassay variability was 6.0% at 28.7 nmol/l and 5.6% at 30.7 nmol/l for 25(OH)D2 and 25(OH)D3, respectively.

### Statistical analysis

Descriptive characteristics of the cohorts are presented as means and standard deviations (SD) when continuous variables were considered, and as frequencies for categorical variables. A generalized linear regression model was used to adjust for age, body mass index (BMI), and sex in the comparison of cases with the control samples. Statistical significance was set at the value of p < 0.05. All statistical analyses were performed using SAS 9.3 (SAS Institute, Cary, NC, USA). This research adhered to guidelines for Strengthening the Reporting of Observational Studies in Epidemiology (STROBE) [21].

### Ethical considerations for human subjects

The protocols for these studies were reviewed and approved by the Institutional Review Board at Loyola University Medical Center, Maywood, IL; the University Hospital of the West Indies/University of the West Indies/Faculty of Medical Sciences Ethics Committee, Mona, Kingston, Jamaica (ECP 06 2009/2010); the Committee on Human Research Publication and Ethics of Kwame Nkrumah University of Science and Technology, Kumasi, Ghana; and the Joint Ethical Committee of the University of Ibadan/University College Hospital, Ibadan, Nigeria. Written informed consent was obtained from all participants.

## Results

Descriptive characteristics of subjects in the present study are presented in Table 1. The number of male subjects is about the same as the number of the female subjects within each of the four study samples. Patients in the SCD cohorts were young adults, had mean ages substantially below the controls in both Jamaica (23.7 ± 2.8 vs. 34.3 ± 6.0 years) and West Africa (23.3 ± 4.4 vs. 34.3 ± 6.7 years). Also, the SCD patients were extremely lean, they were on average 7.2 kg/m^2^ and 4.7 kg/m^2^ lighter than their corresponding Jamaican and West African population controls, respectively.

**Table 1:** **Descriptive characteristics of study subjects** ^**†**^

	Jamaica		West Africa	
	Sickle cell cases	Controls	Sickle cell cases	Controls
Latitude of country of study subjects	18° 15' North	18° 15' North	Nigeria: 10° 00' North	Ghana: 8° 00' North
Longitude of country of study subjects	77° 30' West	77° 30' West	Nigeria: 8° 00' East	Ghana: 2° 00' West
Sample size	20	459	50	497
Number of females (males)	12 (8)	243 (216)	26 (24)	290 (207)
Age (years)	23.7 ± 2.8^b^	34.3 ± 6.0^a^	23.3 ± 4.4^b^	34.3 ± 6.7^a^
Weight (kg)	54.0 ± 7.8^c^	75.9 ± 16.8	52.4 ± 9.7^c^	63.5 ± 11.5
Height (cm)	166.0 ± 7.4^df^	169.3 ± 9.2^d^	164.1 ± 8.8^ef^	162.6 ± 8.2^ef^
Body mass index (kg/m^2^)	19.5 ± 2.5^g^	26.7 ± 6.4	19.4 ± 3.0^g^	24.1 ± 4.5
Vitamin D (nmol/l)	45.4 ± 16.7^h^	72.2 ± 17.8	46.5 ± 12.8^h^	75.9 ± 17.2

^†^Values are expressed as means ± standard deviations; Values with one or two same superscript letters from ‘a’ to ‘h’ are pairwise non-significantly different (*P < 0.05*); vitamin D comparison was adjusted for sex, age and body mass index; All controls were sampled from 2010–2011 and sickle cell cases from 2000–2010 (Jamaica) and 2011–2012 (West Africa).

**Figure 1:**
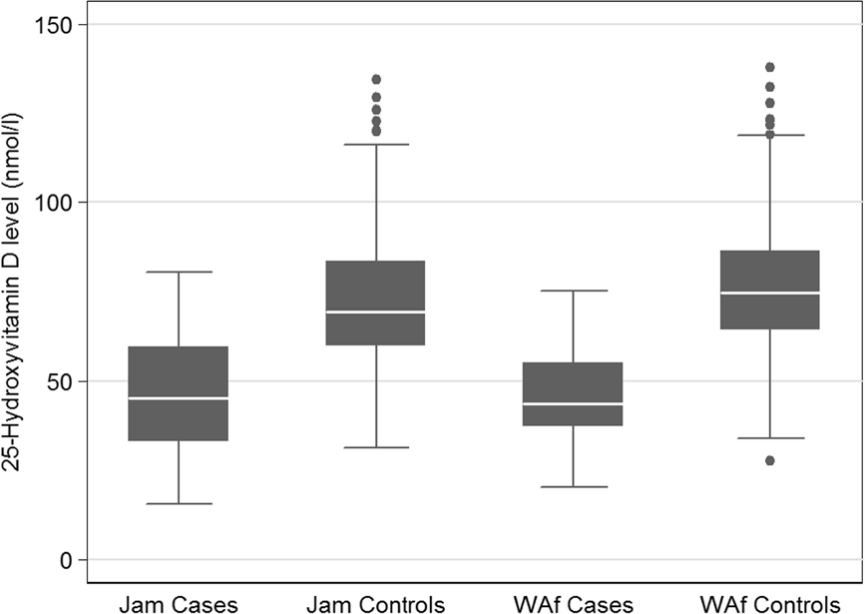
**Box plot of 25(OH)D levels in sickle cell disease cases and controls from Jamaica (Jam) and West Africa (WAf).**

Figure 1 presents a scatter plot of 25(OH)D values in the four study samples. Despite some overlap, the results clearly separated into two distinct distributions. There were a few outlier values among the control study samples. The mean levels of 25(OH)D were lower in SCD patients than in controls in either Jamaica (45.4 ± 16.7 vs. 72.2 ± 17.8 nmol/l) or West Africa (46.5 ± 12.8 vs. 75.9 ± 17.2 nmol/l) after covariate adjustment for sex, age and BMI (Table 1). Even when outlier 25(OH)D values in controls were excluded from analyses, mean 25(OH)D levels for SCD patients were still significantly (*p* < 0.05) lower than those of their corresponding population controls. Compared across the two geographical regions, the Jamaican SCD cohort was not significantly different from the West African SCD cohort in mean age, weight, BMI or levels of plasma 25(OH)D. Similar pattern was observed when the population controls were compared across the two regions (Table 1). For the purpose of comparison, representative data on vitamin D in SCD patients from North American studies [4, 22] are presented in Table 2.

**Table 2:** **Vitamin D in sickle cell disease patients in North America**

Study	Geographic location	Population		Vitamin D levels: 25(OH)D ^†^	
		Cases	Controls	Cases	Controls
Rovner *et al*. [4]	Philadelphia, PA	N:61 SCD-SS	N:89 African-American	37 nmol/l (IQR: 25–82)	52 nmol/l (IQR: 37–90)
	(latitude: 39.95°)	Ages: 5-18	Ages: 6-18		
Buison *et al*. [22]	Philadelphia, PA	N:61 SCD-SS	N:33 African American	Total population: 25.5 ± 12.7 nmol/l	African American: 60.7 ± 21.2 nmol/l
	(latitude: 39.95°)	Ages: 5-18	N:76 Non-African American	Ages: 7–10: N:25 29.5 ± 15.5 nmol/l	Non-African American: 84.6 ± 23.5 nmol/l
			Ages: 7-10		

^†^25(OH)D Measurements: Rovner *et al.*: RIA kit (DiaSorin); Buison *et al.*: Radioiodinated tracer.

N, number; IQR, interquartile range.

We also investigated possible correlations between 25(OH)D levels and laboratory measures of biomarkers of renal or liver function (creatinine, urea, aspartate transaminase and alanine transaminase) among the West African SCD patients for whom these measures were available. No relationship was observed between 25(OH)D levels and the biomarkers (*r* < 0.2, *p* > 0.1).

## Discussion

In this report we confirm that across a wide range of latitudes patients with SCD have low circulating 25(OH)D relative to control populations in the same geographic region. As noted, stored samples separated by an average of 10 years in Jamaica were analyzed and found to be very stable. We can assume, therefore, that these cross-sectional data are representative of a chronic state. In a previous study of samples of 200 healthy Nigerian women that were age-matched to samples of 200 healthy African American women from metropolitan Chicago, we observed that only a small proportion of 25(OH)D distributions in the 2 populations overlapped [23], thus demonstrating the sizeable effect of latitude. Also, the difference in mean levels between these two populations of shared ancestry (African Americans vs. Nigerians) is essentially the same as what is observed between SCD cases and controls in the same geographic location. Representative data from North American studies [4, 22] (Table 2) demonstrate low 25(OH)D among SCD patients just as observed in the current study. Given the findings from Nigeria, and the evidence that 25(OH)D among healthy Nigerians are at the same levels as found among light-skinned European and North American populations [23], we suggest that neither inherent inability to synthesize active 25(OH)D nor limited sun exposure as a result of reduced time spent outdoors can explain the US findings. The great preponderance of 25(OH)D is produced through exposure to UVB radiation so it is also unlikely that inadequate absorption of dietary vitamin D is the key mechanism.

Investigators in Atlanta reported complete resolution of chronic pain in a 16 year old female with SCD who had severe osteoporosis with vitamin D supplementation [2]; serum 25(OH)D levels were reported to be 20 nmol/l [2] at baseline. Bone density improved slightly (+11%) during the 20 months of follow up, and - surprisingly - chronic headaches also resolved [2]. A small scale randomized trial by the same investigators likewise showed a significant improvement in the number of days with pain in addition to physical-activity-related quality of life scores after a short course of high dose vitamin D therapy given as oral cholecalciferol [7]. Thirty-nine patients were randomized, however only 25 were available for end point analysis [7]. Serum 25(OH)D rose significantly in the initial phases of the trial and lower numbers of pain days were reported in the treatment group, although the improvement was not sustained [7]. The authors hypothesize that lower 25(OH)D in SCD could be a result of a combination of lower intake of vitamin D foods, reduced sun exposure and increased utilization of vitamin D for bone remodeling [7]. It seems unlikely, however, that the first two proposed explanations accounted for the low levels of vitamin D observed in the Jamaican and Nigerian sickle cell cohorts, relative to the general population as intake of foods high in vitamin D is universally low [24, 25] and sun exposure is extensive in these populations.

The potential health effects of low 25(OH)D have been widely studied [26–30]. Despite claims from observational studies of a role in conditions varying from cancer to obesity, the accumulated evidence was thought to be inadequate to serve as the basis for supplementation in a recent Institute of Medicine report [1]. In a comprehensive literature review, Aloia argues forcefully that there is no evidence to support health-related consequences of relatively low 25(OH)D beyond bone health [31]. The strongest claim for an etiologic role for 25(OH)D has been made in regard to multiple sclerosis (MS), where month of conception – which in turn reflects circulating 25(OH)D in the mother – has been consistently shown to be associated with this condition in adult offspring [32]. Supplementation to prevent MS is however not currently accepted practice.

Of additional interest in relation to 25(OH)D in SCD patients in tropical countries would be the potential role in mediating immune function [33]. It is interesting to place the relative deficiency observed for patients with SCD in the tropics in the context of global patterns of 25(OH)D [34, 35]. As noted above, SCD patients in Nigeria and Jamaica have similar levels of 25(OH)D to those found among healthy African Americans [36]. As Aloia has pointed out, there are likely to be compensatory mechanisms within the metabolic pathway for 25(OH)D, particularly in the kidney, that give the system overall sufficient plasticity to maintain normal function in the face of “low” circulating 25(OH)D levels [31]. Nonetheless, placing the findings among SCD patients within this context does little to explain why circulating 25(OH)D should be lower than in controls, and nor does it entirely resolve the question of whether there could be pathologic consequences.

## Conclusion

In conclusion, we have demonstrated that circulating 25(OH)D is lower in patients with SCD from equatorial regions to temperate latitudes. This finding, which appears to be characteristic of SCD, could offer a window on vitamin D metabolism if the entirety of the metabolic pathway could be described. In particular, we hypothesize that metabolic demands on the liver in SCD may have reduced the capacity to synthesize vitamin D binding protein. Whether benefits would accrue from supplementation can only be answered with sufficiently large randomized trials where patients are randomized to vitamin D or placebo.

## Abbreviations

25(OH)D: 25-hydroxyvitamin D
BMI: Body mass index
SCD: Sickle cell disease
SD: Standard deviation
SRM: Standard reference material
USA: United States of America
UVB: Ultraviolet B.
